# Prevalence of Self‐Reported Non‐Coeliac Gluten Sensitivity and Its Association With Disorders of Gut‐Brain Interaction and Disordered Eating

**DOI:** 10.1002/ueg2.70256

**Published:** 2026-07-03

**Authors:** Mohamed G. Shiha, David S. Sanders, Helen Burton‐Murray, Magnus Simren, Olafur Palsson, Imran Aziz

**Affiliations:** ^1^ Division of Clinical Medicine School of Medicine and Population Health University of Sheffield Sheffield UK; ^2^ Academic Unit of Gastroenterology Sheffield Teaching Hospitals Sheffield UK; ^3^ Department of Medicine Division of Gastroenterology Massachusetts General Hospital Boston Massachusetts USA; ^4^ Harvard Medical School Boston Massachusetts USA; ^5^ Department of Molecular and Clinical Medicine Institute of Medicine Sahlgrenska Academy University of Gothenburg Gothenburg Sweden; ^6^ Center for Functional GI and Motility Disorders University of North Carolina at Chapel Hill Chapel Hill North Carolina USA

**Keywords:** avoidant/restrictive food intake disorder, brain‐gut axis, disorders of gut‐brain interaction, feeding and eating disorders, gluten, noncoeliac gluten sensitivity, sensitivity

## Abstract

**Background:**

Noncoeliac gluten sensitivity (NCGS) remains a controversial clinical entity at the intersection between disorders of gut–brain interaction (DGBI) and disordered eating. We aimed to determine the prevalence of self‐reported NCGS and to characterise its association with DGBI and avoidant/restrictive food intake disorder (ARFID) symptoms in an adult general population.

**Methods:**

We conducted a population‐based internet survey with pre‐defined demographic quotas across the United States of America and United Kingdom in 2023. Participants completed the Rome IV diagnostic questionnaire, the Nine‐Item ARFID screen, and validated instruments for psychological distress, somatisation and quality of life.

**Results:**

A total of 4002 participants (50% female; median age 46 years) were included in the analyses. The prevalence of NCGS was 14.2% (95% CI, 13.1–15.3). Participants with self‐reported NCGS reported more nongluten food intolerances than those without self‐reported NCGS (median 3 vs. 0, *p* < 0.001). Among individuals with NCGS, 69.4% (95% CI, 65.4–73.1) had concomitant DGBI and/or ARFID symptoms, with nearly one‐quarter (24.0%; 95% CI, 20.6–27.8) meeting the criteria for all three conditions. Those with comorbid self‐reported NCGS, DGBI and ARFID symptoms had the highest levels of psychological distress, somatic symptom reporting, increased healthcare utilisation and reduced quality of life (all *p* < 0.001).

**Conclusion:**

NCGS is reported by approximately one in seven adults in the United States of America and United Kingdom. Individuals with self‐reported NCGS frequently meet diagnostic criteria for DGBI and/or ARFID symptoms, and those who experience all three entities represent a distinct high‐severity phenotype. Our findings suggest that self‐reported NCGS may represent a broader syndrome of food‐related symptom attribution rather than gluten‐specific pathology.

## Key Summary

- What is the established knowledge on this subject?
  ◦ Noncoeliac gluten sensitivity (NCGS) is a condition characterised by gastrointestinal and extra‐intestinal symptoms following gluten ingestion in individuals without coeliac disease.
  ◦ It is currently unclear whether NCGS represents a specific food hypersensitivity or a more heterogeneous entity driven by overlapping disorders of gut‐brain interaction (DGBI) and disordered eating, such as avoidant/restrictive food intake disorder (ARFID).
- What are the significant and/or new findings of this study?
  ◦ This study provides the first large‐scale evaluation of the epidemiology and clinical associations of self‐reported NCGS using a rigorous, standardised methodology across the United States of America and United Kingdom.
  ◦ NCGS is reported by 14.2% of adults in the United States of America and United Kingdom. Self‐reported NCGS is strongly associated with DGBI across all gastrointestinal regions and with concurrent ARFID symptoms.
  ◦ The burden of these overlapping comorbidities is the primary driver of symptom severity, psychological distress, healthcare utilisation and impaired quality of life.
  ◦ Most individuals with self‐reported NCGS report adverse reactions to multiple nongluten foods and do not follow a gluten‐free diet.

## 1 Introduction

Non‐coeliac gluten sensitivity (NCGS) is a common yet poorly defined clinical entity characterised by gastrointestinal and extra‐intestinal symptoms triggered by the ingestion of gluten in individuals without coeliac disease [1]. NCGS is reported by approximately 10% of the general population worldwide, with significant geographical variations [2]. However, these prevalence estimates are limited by substantial heterogeneity across studies, likely due to inconsistent diagnostic criteria, residual confounding factors, and true population differences [2].

Unlike coeliac disease, NCGS lacks validated biomarkers and is solely defined by subjective patient reporting. Experts have proposed a double‐blind, placebo‐controlled gluten challenge (the Salerno protocol) as a potential diagnostic “gold standard” [3]. However, this approach is complex, time‐consuming, and impractical for routine clinical care. Moreover, randomised controlled trials have consistently shown a substantial nocebo effect, where symptoms are triggered by negative expectancy rather than gluten itself [4, 5]. High‐quality crossover data further demonstrate the strengths of these perceptions, as many individuals with self‐reported NCGS choose to maintain a gluten‐free diet despite being unblinded to results confirming that their symptomatic response to gluten was indistinguishable from placebo [6].

NCGS demonstrates significant overlap with irritable bowel syndrome (IBS), including shared psychological features of anxiety and depression, prompting our recent proposal to reclassify it within the spectrum of disorders of gut‐brain interactions (DGBI) [2, 7]. However, while the link to IBS is established, the association between self‐reported NCGS and the broader range of other DGBI remains unknown. Moreover, the avoidant eating patterns and multiple food restrictions commonly reported in self‐reported NCGS and in DGBI raise untested questions about an overlap with disordered eating, including avoidant/restrictive food intake disorder (ARFID). We therefore aimed to evaluate the prevalence of self‐reported NCGS in the general population, characterise its association with DGBI and disordered eating, and quantify how these comorbidities impact clinical disease burden.

## 2 Methods

### 2.1 Study Design and Setting

We conducted a cross‐sectional, population‐based internet survey across the United States (USA) and the United Kingdom (UK) from October to December 2023. Participants were recruited nationwide in each country through Qualtrics Inc. (Provo, Utah, USA), using predefined age (40% aged 18–39 years, 40% aged 40–64 years and 20% aged 65 years and older) and sex (50% male; 50% female) quotas to ensure samples were representative of the general population in each country. Our internet survey used the same approach as the Rome Foundation Global Epidemiological Survey [8]. The survey was sent to the invited participants until the required age and sex quotas were reached.

### 2.2 Participants

Eligible participants were adults aged 18 years and older residing in the USA or the UK. To minimise selection bias, potential respondents were invited to complete a “general health survey” without specific reference to gluten sensitivity, gastrointestinal symptoms, or eating disorder symptoms. Strict quality assurance measures were applied, including restricting responses to one per device and excluding participants who failed attention checks or demonstrated excessive inconsistency across repeated diagnostic questions. Informed electronic consent was obtained from all participants before data collection. No personally identifiable data were collected.

### 2.3 Measures and Definitions

The survey collected data on demographics and medical history. Participants were asked about their history of organic gastrointestinal disease (e.g., coeliac disease, diverticulitis, peptic ulcer disease, inflammatory bowel disease or gastrointestinal cancer); those reporting such diagnoses were excluded from classification as having a DGBI. Information on healthcare utilisation was also collected, including medication use and history of abdominal surgery.

We collected detailed data on dietary patterns, including the presence of specific food intolerances, and following dietary restrictions. NCGS was defined as self‐reported intolerance to gluten in the absence of a self‐reported history of medical diagnosis of coeliac disease. Participants reporting coeliac disease were excluded from all analyses involving NCGS.

We assessed clinical variables and patient‐reported outcomes using several validated instruments. We identified and categorised DGBI using the complete Rome IV diagnostic questionnaire for adults [9]. We used the Nine‐Item ARFID screen (NIAS) [10] for restrictive eating behaviour and the SCOFF questionnaire [11] to screen for eating disorders. We evaluated psychological distress using the Patient Health Questionnaire‐4 (PHQ‐4) [12] and assessed the burden of extra‐intestinal somatic symptoms using the modified PHQ‐12 [13]. We evaluated health‐related quality of life using the PROMIS Global‐10 instrument [14]. Detailed descriptions of all instruments are provided in the Supplementary Material.

### 2.4 Ethical Approval

Before data collection started, the study was reviewed by the Institutional Review Board (IRB) at the University of North Carolina (USA) and the University of Sheffield (UK). It was deemed IRB exempt because all study participants were anonymous to the investigators.

### 2.5 Statistical Analyses

Descriptive statistics for categorical variables were presented as raw numbers and percentages, while continuous variables were summarised as medians with interquartile ranges (IQR). Prevalence was reported as percentages with 95% confidence intervals (CI) based on the binomial distribution. Continuous variables were compared between two groups using the Mann‐Whitney *U* test and across multiple groups using the Kruskal‐Wallis test. Categorical variables were compared using the Chi‐square test. A *p*‐value of < 0.05 was considered statistically significant. Associations between self‐reported NCGS, DGBI and disordered eating were assessed using univariable and multivariable logistic regression models. Results were reported as odds ratios (OR) and adjusted OR (aOR) with 95% CI. All analyses were conducted using StataNow version 19.5 (StataCorp, College Station, TX, USA).

## 3 Results

### 3.1 Baseline Characteristics

A total of 4002 participants completed the survey (USA, *n* = 2000; UK, *n* = 2002). The cohort had a median age of 46 years (IQR 33–62) and was 50.0% female. The median BMI was 26.6 (23.1–31.3), and 81.1% were white (Table 1). The baseline characteristics were similar across the USA and UK, as shown in Table S1.

**TABLE 1:** Characteristics of study participants.

	Total (*n* = 4002)	NCGS (*n* = 569)	No NCGS (*n* = 3400)	OR (95% CI)	Adjusted OR^a^ (95% CI)
Demographic characteristics					
Country, *n* (%)					
USA	2000 (50)	271 (47.6)	1712 (50.3)	—	—
UK	2002 (50)	298 (52.3)	1688 (49.6)	—	—
Female sex, *n* (%)	2000 (50)	312 (54.8)	1668 (49.0)	1.26 (1.05–1.50)	—
White ethnicity, *n* (%)	3246 (81.1)	430 (75.5)	2784 (81.8)	0.68 (0.55–0.84)	—
Age, median (IQR)	46 (33–62)	39 (29–54)	47 (34–63)	—	—
BMI, median (IQR)	26.6 (23.1–31.3)	27.2 (22.8–32.1)	26.6 (23.1–31.2)	—	—
Gastrointestinal symptoms, *n* (%)^b^					
Postprandial fullness	701 (17.5)	150 (26.4)	544 (16.0)	1.87 (1.52–2.31)	1.65 (1.33–2.04)
Dyspepsia	258 (6.4)	71 (12.5)	181 (5.3)	2.53 (1.89–3.38)	2.24 (1.67–3.01)
Nausea	279 (7.0)	75 (13.2)	200 (6.0)	2.42 (1.83–3.21)	2.06 (1.55–2.75)
Bloating or distention	918 (23.0)	235 (41.3)	668 (19.6)	2.87 (2.38–3.46)	2.64 (2.18–3.21)
Abdominal pain	513 (12.8)	128 (22.5)	374 (11.0)	2.34 (1.87–2.93)	2.12 (1.68–2.66)
Constipation	1128 (28.2)	228 (40.1)	887 (26.1)	1.89 (1.57–2.27)	1.65 (1.36–1.99)
Diarrhoea	1008 (25.2)	219 (38.5)	774 (22.8)	2.12 (1.76–2.55)	1.93 (1.59–2.33)
Extra‐intestinal symptoms, *n* (%)^c^					
Back pain	885 (22.1)	143 (25.1)	737 (21.7)	1.21 (0.98–1.49)	1.17 (0.95–1.44)
Arthralgia	789 (19.7)	129 (22.7)	653 (19.2)	1.23 (0.99–1.52)	1.32 (1.06–1.65)
Headache	494 (12.3)	99 (17.4)	389 (11.4)	1.63 (1.28–2.07)	1.35 (1.06–1.73)
Chest pain	110 (2.7)	25 (4.4)	84 (2.5)	1.81 (1.15–2.86)	1.50 (0.95–2.39)
Dizziness	195 (5.0)	57 (10.0)	134 (3.9)	2.71 (1.96–3.75)	2.32 (1.67–3.23)
Fainting spells	56 (1.4)	10 (1.8)	44 (1.3)	1.36 (0.68–2.72)	1.11 (0.55–2.24)
Palpitations	220 (5.5)	58 (10.2)	159 (4.7)	2.31 (1.68–3.16)	1.95 (1.41–2.69)
Shortness of breath	263 (6.6)	52 (9.1)	210 (6.2)	1.52 (1.11–2.09)	1.43 (1.04–1.98)
Fatigue	1164 (29.1)	220 (38.7)	930 (27.4)	1.67 (1.39–2.01)	1.44 (1.18–1.74)
Insomnia	1068 (26.7)	196 (34.4)	863 (25.4)	1.54 (1.27–1.86)	1.38 (1.14–1.68)
Psychological burden and quality of life, *n* (%)					
Anxiety	1065 (26.6)	212 (37.3)	841 (24.7)	1.80 (1.49–2.17)	1.52 (1.25–1.84)
Depression	1032 (25.8)	203 (35.7)	819 (24.1)	1.74 (1.44–2.11)	1.51 (1.24–1.83)
High somatisation	301 (7.5)	80 (14.1)	218 (6.4)	2.38 (1.81–3.13)	1.95 (1.47–2.58)
Stress related to GI function	486 (12.1)	126 (22.1)	352 (10.4)	1.83 (1.62–2.07)	1.70 (1.50–1.93)
Concern about bowel symptoms	464 (11.6)	111 (19.5)	345 (10.1)	1.64 (1.44–1.85)	1.57 (1.38–1.78)
Below‐average mental quality of life	2668 (66.7)	416 (73.1)	2226 (65.5)	1.43 (1.17–1.74)	1.23 (1.00–1.51)
Below‐average physical quality of life	2601 (65.0)	403 (70.8)	2171 (64.0)	1.37 (1.13–1.66)	1.34 (1.10–1.63)

*Note:* The total counts include 4002 participants, while the NCGS/No NCGS comparison excludes those with self‐reported coeliac disease (*n* = 33).

^a^ Adjusted for age, sex, and ethnicity.

^b^ Gastrointestinal symptoms were assessed using the Rome IV diagnostic questionnaire.

^c^ Somatic symptoms were assessed using the PHQ‐12, defined as “bothered a lot”.

### 3.2 Prevalence and Burden of Self‐Reported NCGS

The overall prevalence of coeliac disease was 0.82% (95% CI, 0.57–1.16). In contrast, the prevalence of self‐reported NCGS in the total population was 14.2% (95% CI, 13.1–15.3), with similar prevalence observed in the USA (13.5%; 95% CI, 12.0–15.1) and UK (14.8%; 95% CI, 13.3–16.5). Compared with individuals without gluten sensitivity, those with self‐reported NCGS were younger (median age 39 vs. 47 years; *p* < 0.001), more likely to be female (OR 1.26; 95% CI, 1.05–1.50), and less likely to be of White ethnicity (OR 0.68; 95% CI, 0.55–0.84) (Table 1).

The self‐reported NCGS cohort reported significantly higher prevalence of all assessed gastrointestinal symptoms than those not reporting gluten sensitivity. After adjusting for age, sex, and ethnicity, the strongest associations were observed for bloating (41.3% vs. 19.6%; aOR 2.64; 95% CI, 2.18–3.21), dyspepsia (12.5% vs. 5.3%; aOR 2.24; 95% CI, 1.67–3.01), and abdominal pain (22.5% vs. 11.0%; aOR 2.12; 95% CI, 1.68–2.66).

Individuals with self‐reported NCGS were also more likely to report extra‐intestinal somatic symptoms than those without gluten sensitivity. Fatigue was the most prevalent complaint, reported by 38.7% of the self‐reported NCGS group and 27.4% of the non‐NCGS group (aOR 1.44; 95% CI, 1.18–1.74). Moreover, individuals with self‐reported NCGS had higher frequencies of screening positive for anxiety (aOR 1.52; 95% CI, 1.25–1.84), depression (aOR 1.51; 95% CI, 1.24–1.83), and high somatisation (aOR 1.95; 95% CI, 1.47–2.58). Perceived health status was markedly impaired in individuals with self‐reported NCGS, with higher odds of reporting below‐average physical (aOR 1.34; 95% CI, 1.10–1.63) and mental (aOR 1.23; 95% CI, 1.00–1.51) quality of life.

### 3.3 Association Between Self‐Reported NCGS and DGBI

The overall prevalence of DGBI across the USA and the UK was 42.6% (95% CI, 41.0–44.1). After adjusting for demographics and mood disorders, individuals with self‐reported NCGS had a significantly higher odds of fulfilling the Rome IV diagnostic criteria for a DGBI compared with those without self‐reported NCGS (58.7% vs. 40.3%; aOR 1.80; 95% CI, 1.49–2.18) (Table 2). This association was observed across all anatomical DGBI regions but was most pronounced for gastroduodenal (aOR 1.70; 95% CI, 1.37–2.11) and bowel (aOR 1.67; 95% CI, 1.38–2.02) disorders. Because of the small number of cases, we excluded centrally mediated disorders of gastrointestinal pain (*n* = 1) and biliary disorders (*n* = 7) from analyses. Self‐reported NCGS was strongly associated with painful DGBI, including functional heartburn (aOR 1.87; 95% CI, 1.19–2.92), functional dyspepsia (aOR 1.74; 95% CI, 1.37–2.21), and IBS (aOR 1.93; 95% CI, 1.42–2.62). Functional abdominal bloating and distention was also strongly associated with self‐reported NCGS (aOR 2.12; 95% CI, 1.44–3.12). Conversely, other painless DGBIs, such as functional constipation (aOR 1.12; 95% CI, 0.83–1.51) and functional diarrhoea (aOR 1.10; 95% CI, 0.76–1.61), did not have a significant association with self‐reported NCGS.

**TABLE 2:** Association between NCGS and DGBI.

	Total (*n* = 4002)	NCGS (*n* = 569)	No NCGS (*n* = 3400)	OR (95% CI)	Adjusted OR^a^ (95% CI)
Any DGBI, *n* (%)	1704 (42.6)	334 (58.7)	1370 (40.3)	2.10 (1.75–2.52)	1.80 (1.49–2.18)
Any oesophageal DGBI, *n* (%)	404 (10.1)	87 (15.3)	317 (9.3)	1.75 (1.35–2.26)	1.51 (1.16–1.97)
Functional heartburn, *n* (%)	110 (2.7)	28 (4.9)	82 (2.4)	2.09 (1.35–3.24)	1.87 (1.19–2.92)
Functional chest pain, *n* (%)	81 (2.0)	19 (3.3)	62 (1.8)	1.85 (1.10–3.13)	1.52 (0.89–2.59)
Reflux hypersensitivity, *n* (%)	68 (1.7)	12 (2.1)	56 (1.6)	1.28 (0.68–2.41)	1.11 (0.58–2.10)
Globus, *n* (%)	33 (0.8)	4 (0.7)	29 (0.9)	0.82 (0.28–2.34)	0.93 (0.32–2.69)
Functional dysphagia, *n* (%)	227 (5.7)	51 (9.0)	176 (5.2)	1.80 (1.30–2.49)	1.53 (1.10–2.14)
Any gastroduodenal DGBI, *n* (%)	655 (16.4)	151 (26.5)	504 (14.8)	2.07 (1.68–2.55)	1.70 (1.37–2.11)
Functional dyspepsia	475 (12.0)	115 (20.2)	360 (10.6)	2.13 (1.69–2.69)	1.74 (1.37–2.21)
Postprandial distress syndrome	418 (10.4)	97 (17.0)	321 (9.4)	1.97 (1.53–2.52)	1.59 (1.23–2.05)
Epigastric pain syndrome	169 (4.2)	47 (8.3)	122 (3.6)	2.41 (1.70–3.4)	1.94 (1.36–2.78)
Belching disorders	40 (1.0)	12 (2.1)	28 (0.8)	2.59 (1.31–5.13)	2.09 (1.04–4.18)
Nausea and vomiting disorders	150 (3.7)	33 (5.8)	117 (3.4)	1.72 (1.16–2.56)	1.27 (0.85–1.91)
Chronic nausea and vomiting	81 (2.0)	15 (2.6)	66 (1.9)	1.36 (0.77–2.41)	0.97 (0.54–1.74)
Cyclical vomiting syndrome	73 (1.8)	12 (2.1)	61 (1.8)	1.17 (0.63–2.20)	0.92 (0.48–1.73)
Cannabinoid hyperemesis syndrome	16 (0.4)	8 (1.4)	8 (0.2)	6.04 (2.26–16.1)	3.82 (1.41–10.34)
Any bowel DGBI, *n* (%)	1302 (32.5)	263 (46.2)	1039 (30.6)	1.95 (1.63–2.33)	1.67 (1.38–2.02)
Irritable bowel syndrome (IBS)	242 (6.0)	68 (12.0)	174 (5.1)	2.51 (1.87–3.38)	1.93 (1.42–2.62)
IBS with predominant constipation	79 (2.0)	24 (4.2)	55 (1.6)	2.67 (1.64–4.36)	1.99 (1.21–3.29)
IBS with predominant diarrhoea	67 (1.7)	23 (4.0)	44 (1.3)	3.21 (1.92–5.36)	2.58 (1.53–4.36)
IBS with mixed bowel habits	88 (2.2)	20 (3.5)	68 (2.0)	1.78 (1.07–2.96)	1.31 (0.78–2.21)
IBS unclassified	8 (0.2)	1 (0.2)	7 (0.2)	0.85 (0.10–6.94)	0.69 (0.08–5.84)
Functional constipation	360 (9.0)	62 (11.0)	298 (8.8)	1.27 (0.95–1.69)	1.12 (0.83–1.51)
Functional diarrhoea	219 (5.5)	36 (6.3)	183 (5.4)	1.18 (0.82–1.71)	1.10 (0.76–1.61)
Functional abdominal bloating/distension	143 (3.6)	39 (7.0)	104 (3.1)	2.33 (1.59–3.40)	2.12 (1.44–3.12)
Unspecified functional bowel disorder	300 (7.5)	52 (9.1)	248 (7.3)	1.27 (0.93–1.74)	1.17 (0.85–1.62)
Opioid‐induced constipation	64 (1.6)	10 (1.8)	54 (1.6)	1.1 (0.56–2.18)	1.03 (0.52–2.06)
Any anorectal DGBI, *n* (%)	364 (9.1)	78 (13.7)	286 (8.4)	1.72 (1.32–2.26)	1.48 (1.12–1.95)
Faecal incontinence	125 (3.1)	26 (4.6)	99 (2.9)	1.59 (1.02–2.48)	1.59 (1.01–2.51)
Levator ani syndrome	77 (1.9)	17 (3.0)	60 (1.8)	1.71 (0.99–2.95)	1.30 (0.74–2.27)
Proctalgia fugax	214 (5.3)	47 (8.3)	167 (5.0)	1.74 (1.24–2.44)	1.44 (1.02–2.04)

*Note:* The total counts include 4002 participants, while the NCGS/No NCGS comparison excludes those with self‐reported coeliac disease (*n* = 33).

^a^ Adjusted for age, sex, ethnicity, and mood disorders.

### 3.4 Association Between Self‐Reported NCGS and Disordered Eating

A total of 1035 individuals were screened positive for ARFID symptoms (25.8%; 95% CI, 24.5–27.2). Individuals with self‐reported NCGS were more likely to screen positive for ARFID symptoms compared with those without self‐reported NCGS (34.8% vs. 24.3%; aOR 1.46; 95% CI, 1.20–1.77) (Table 3). Self‐reported NCGS was associated with all three ARFID symptoms phenotypes, but the strongest association was observed with a positive screen for fear of aversive consequences (aOR 2.56; 95% CI, 1.91–3.43). Similarly, individuals with self‐reported NCGS generally demonstrated a higher frequency of elevated disordered eating symptoms. A positive SCOFF screen was more common in the self‐reported NCGS cohort compared with the non‐NCGS cohort (31.5% vs. 16.0%; aOR 1.99; 95% CI, 1.62–2.45).

**TABLE 3:** Association between NCGS, disordered eating, and dietary patterns.

	Total (*n* = 4002)	NCGS (*n* = 569)	No NCGS (*n* = 3400)	OR (95% CI)	Adjusted OR^a^ (95% CI)
Positive ARFID symptoms screen, *n* (%)	1035 (25.8)	198 (34.8)	826 (24.3)	1.66 (1.37–2.01)	1.46 (1.20–1.77)
ARFID symptoms phenotypes, *n* (%)					
Sensory‐based avoidance	557 (13.9)	106 (18.6)	446 (13.1)	1.51 (1.20–1.91)	1.31 (1.03–1.66)
Lack of interest in eating/appetite	612 (15.3)	121 (21.3)	482 (14.2)	1.63 (1.30–2.04)	1.44 (1.15–1.81)
Fear of aversive consequences	247 (6.2)	75 (13.2)	168 (5.0)	2.92 (2.18–3.89)	2.56 (1.91–3.43)
Positive SCOFF screen, *n* (%)	728 (18.2)	179 (31.5)	541 (16.0)	2.42 (1.98–2.96)	1.99 (1.62–2.45)
Individual SCOFF items, *n* (%)					
Sick: Made yourself sick because you felt uncomfortably full?	319 (8.0)	88 (15.5)	227 (6.7)	2.55 (1.96–3.33)	2.03 (1.54–2.67)
Control: Worried you have lost control over how much you eat?	678 (17.0)	157 (27.6)	516 (15.2)	2.12 (1.73–2.61)	1.73 (1.39–2.14)
One stone: Lost more than one stone (6.35 kg) in 3 months?	471 (11.8)	96 (17.0)	367 (10.8)	1.67 (1.31–2.14)	1.55 (1.21–1.99)
Fat: Believe yourself to be fat when others say you are too thin?	588 (14.7)	131 (23.0)	453 (13.3)	1.94 (1.56–2.42)	1.64 (1.31–2.05)
Food: Would you say food dominates your life?	582 (14.5)	136 (24.0)	438 (13.0)	2.12 (1.70–2.63)	1.76 (1.41–2.21)
Self‐reported food sensitivity, *n* (%)					
Dairy products	1009 (25.2)	331 (58.2)	670 (19.7)	5.66 (4.70–6.83)	5.17 (4.27–6.25)
Soy	318 (8.0)	168 (29.5)	139 (4.1)	9.82 (7.67–12.58)	9.63 (7.47–12.40)
Eggs	480 (12.0)	224 (39.4)	254 (7.5)	8.04 (6.5–9.93)	7.47 (6.03–9.26)
Fish	320 (8.0)	156 (27.4)	157 (4.6)	7.80 (6.11–9.95)	7.94 (6.17–10.21)
Nuts	410 (10.2)	183 (32.2)	220 (6.5)	6.85 (5.48–8.56)	7.30 (5.81–9.18)
Onions	497 (12.4)	206 (36.2)	280 (8.2)	6.32 (5.12–7.80)	6.76 (5.44–8.39)
Sugary foods	589 (14.7)	259 (45.5)	322 (9.5)	7.98 (6.53–9.76)	7.44 (6.0–9.11)
Dietary restrictions, *n* (%)					
Gluten‐free diet	113 (2.8)	70 (12.3)	20 (0.6)	23.7 (14.29–39.3)	22.2 (13.3–37.07)
Dairy‐free diet	146 (3.6)	46 (8.1)	96 (2.8)	3.0 (2.10–4.35)	2.62 (1.81–3.79)
Vegetarian diet	168 (4.2)	48 (8.4)	118 (3.5)	2.56 (1.80–3.62)	2.24 (1.57–3.19)
Vegan diet	63 (1.6)	19 (3.3)	43 (1.3)	2.69 (1.56–4.66)	2.41 (1.38–4.19)
Low‐FODMAP diet	22 (0.5)	10 (1.8)	10 (0.3)	6.06 (2.51–14.63)	5.26 (2.15–12.88)
Low sugar diet	272 (6.8)	50 (8.8)	219 (6.4)	1.39 (1.01–1.92)	1.62 (1.17–2.26)

*Note:* The total counts include 4002 participants, while the NCGS/No NCGS comparison excludes those with self‐reported coeliac disease (*n* = 33).

Abbreviations: FODMAP, fermentable oligosaccharides, disaccharides, monosaccharides and polyols; NCGS, non‐coeliac gluten sensitivity.

^a^ Adjusted for age, sex, and ethnicity.

### 3.5 Dietary Behaviours

Individuals with self‐reported NCGS commonly reported adverse symptoms to multiple non‐gluten food groups (Table 3). Adverse reactions were most frequently reported for dairy products (58.2% vs. 19.7%; aOR 5.17; 95% CI, 4.27–6.25) and sugary foods (45.5% vs. 9.5%; aOR 7.44; 95% CI, 6.0–9.11). Moreover, approximately a third of the self‐reported NCGS cohort reported concurrent sensitivity to eggs (39.4%), onions (36.2%), and nuts (32.2%), with a 6‐ to 7‐ fold increase in odds compared with those without NCGS. Overall, individuals with self‐reported NCGS reported a broader range of dietary sensitivities, with a median of three non‐gluten food sensitivities (IQR 1–6), compared with 0 (IQR 0–2) in the non‐NCGS cohort (*p* < 0.001).

Following a gluten‐free diet was low among individuals with self‐reported NCGS, albeit still significantly higher than those not reporting gluten sensitivity (12.3% vs. 0.6%; aOR 22.2; 95% CI, 13.3–37.07). Individuals with self‐reported NCGS also demonstrated broader restrictive dietary patterns and were significantly more likely than those without self‐reported gluten sensitivity to adopt other exclusion diets, including dairy‐free, low‐FODMAP, low‐sugar, vegan and vegetarian diets.

### 3.6 Comorbidity and Cumulative Burden of Self‐Reported NCGS, DGBI, and ARFID Symptoms

**FIGURE 1:**
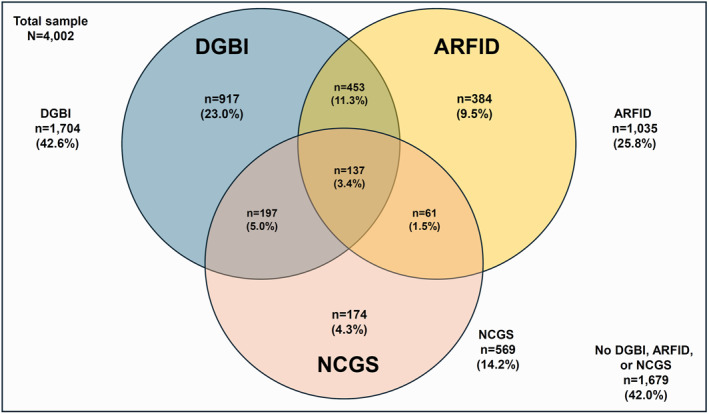
The overlap between self‐reported NCGS, DGBI, and ARFID symptoms within the general adult population of the USA and UK.

**FIGURE 2:**
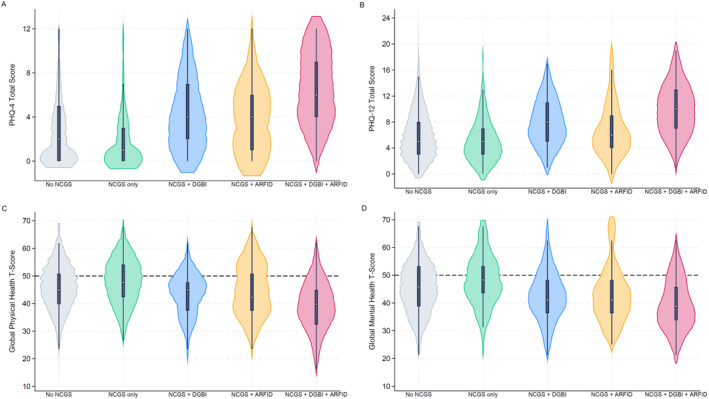
Violin plots illustrating the distribution of psychological distress, somatic symptom burden and quality of life scores across five study groups (A) PHQ‐4 total score for anxiety and depression, (B) PHQ‐12 total score for non‐gastrointestinal somatic symptom burden, (C) PROMIS global physical health T‐scores and (D) PROMIS global mental health T‐scores. The internal markers of each violin indicate the median (white dot) and interquartile range (bar). The width of each violin represents the probability density of the data at different values.

The overlap between self‐reported NCGS, DGBI and ARFID symptoms is shown in Figure 1. Of the 569 individuals with self‐reported NCGS, only 30.6% (*n* = 174) presented with isolated self‐reported NCGS, while the majority fulfilled the criteria for a comorbid DGBI and/or ARFID symptoms. Approximately 1 in 4 people with self‐reported NCGS had concurrent DGBI and ARFID symptoms. Analyses of these comorbid subgroups demonstrated a significant cumulative health burden (Table 4). Individuals with the overlapping triad of self‐reported NCGS, DGBI and ARFID symptoms represented a distinct, high‐severity phenotype with higher psychological distress, somatic symptom reporting, and lower physical and mental quality of life compared with those with NCGS alone (Figure 2). This highly comorbid group was significantly younger (*p* < 0.001) and had the highest prevalence of underweight BMI (6.7%). Conversely, those with self‐reported NCGS alone represented a milder clinical profile characterised by older age, higher BMI and significantly lower frequencies of screening positive for psychological distress and somatisation (Table 4).

**TABLE 4:** Characteristics and healthcare outcomes in individuals with self‐reported NCGS, stratified by the presence of DGBI and ARFID symptoms.

	NCGS alone (*n* = 174)	NCGS + DGBI (*n* = 197)	NCGS + ARFID symptoms (*n* = 61)	NCGS + DGBI + ARFID symptoms (*n* = 137)	*p*‐value
Demographics					
Female sex, *n* (%)	89 (51.1)	121 (61.4)	26 (42.6)	76 (55.5)	0.04
White ethnicity, *n* (%)	142 (81.6)	146 (74.1)	42 (69.0)	100 (73.0)	0.13
Age, median (IQR)	49 (32–61)	39 (30–52)	35 (30–53)	32 (26–46)	< 0.001
BMI categories^a^					
Underweight (< 18.5)	1 (0.8)	3 (2.0)	2 (5.3)	7 (6.7)	0.03
Normal weight (18.5–24.9)	51 (39.0)	47 (30.7)	13 (34.2)	44 (42.3)	
Overweight (25–29.9)	41 (31.3)	42 (27.5)	12 (31.6)	18 (17.3)	
Obese (30+)	38 (29.0)	61 (39.9)	11 (29.0)	35 (33.7)	
Psychological distress and somatisation					
Anxiety	24 (13.8)	81 (41.1)	22 (36.1)	85 (62.0)	< 0.001
Depression	24 (13.8)	73 (37.1)	22 (36.1)	84 (61.3)	< 0.001
High somatisation	9 (5.2)	26 (13.2)	7 (11.5)	38 (27.7)	< 0.001
Stress related to GI function	22 (12.6)	50 (25.4)	11 (18.0)	43 (31.4)	< 0.001
Concern about bowel symptoms	9 (5.2)	40 (20.3)	15 (24.6)	47 (34.3)	< 0.001
Below‐average mental quality of life	100 (57.5)	157 (79.7)	47 (77.0)	112 (81.8)	< 0.001
Below‐average physical quality of life	92 (53.0)	153 (77.7)	37 (60.7)	121 (88.3)	< 0.001
Healthcare utilisation					
Doctor visits (≥ 1 per month)	11 (6.3)	26 (13.2)	15 (24.6)	29 (21.2)	< 0.001
GI‐related healthcare visits	42 (24.1)	91 (46.2)	25 (41.0)	72 (52.6)	< 0.001
GI‐specific medication	57 (32.8)	96 (48.7)	32 (52.5)	91 (66.4)	< 0.001
Analgesics	52 (30.0)	85 (43.1)	32 (52.5)	90 (65.7)	< 0.001
Psychotropic medication	45 (26.0)	81 (41.1)	23 (37.7)	86 (62.8)	< 0.001
Appendectomy	16 (9.2)	16 (8.1)	11 (18.0)	28 (20.4)	0.002
Cholecystectomy	14 (8.0)	15 (7.6)	5 (8.2)	12 (8.8)	0.986
Hysterectomy	12 (7.0)	7 (3.6)	6 (9.8)	15 (11.0)	0.055
Bowel resection	10 (5.7)	1 (0.5)	4 (6.6)	2 (1.5)	0.006
Dietary behaviour					
Gluten‐free diet	16 (9.2)	25 (12.7)	14 (23.0)	15 (11.0)	0.04
Restriction of > 2 distinct food groups	2 (1.1)	6 (3.0)	4 (6.6)	4 (2.9)	0.17

Abbreviations: ARFID, avoidant/restrictive food intake disorder; DGBI, disorders of gut‐brain interaction; GI, gastrointestinal; NCGS, non‐coeliac gluten sensitivity.

^a^ BMI was not available in all participants.

**FIGURE 3:**
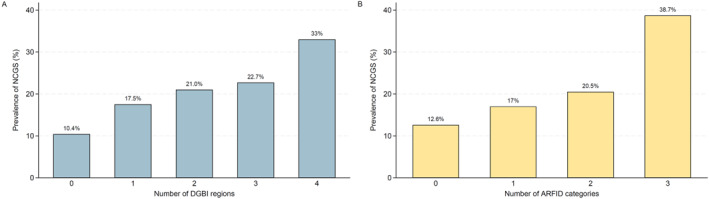
The association between the prevalence of NCGS and the number of DGBI regions (A) and the number of ARFID symptoms categories (B).

Psychological distress followed a clear stepwise increase across the groups. The prevalence of clinically significant anxiety and depression was only 13.8% in the self‐reported NCGS alone group, which is lower than the frequencies observed in the overall population. However, this significantly increased with concurrent DGBI or ARFID symptoms and rose to over 60% in the group with the overlapping triad of self‐reported NCGS, DGBI, and ARFID symptoms. The burden of somatic symptoms, stress‐related to gastrointestinal function and concern about bowel symptoms were also highest in the triad group. Furthermore, the prevalence of self‐reported NCGS increased as the number of anatomical regions affected by DGBI and the number of ARFID symptoms categories increased (Figure 3).

While the self‐reported NCGS alone group reported minimal engagement with healthcare services and average medication use, the overlap with DGBI and/or ARFID symptoms was associated with significantly higher rates of monthly doctor visits and medication use.

Following a gluten‐free diet was reported more frequently in the comorbid ARFID symptoms group (23%), who also reported higher rates of broader dietary avoidance (6.6%) (Table 4).

## 4 Discussion

In this population‐based study, we found that self‐reported NCGS is a highly prevalent complaint, affecting 14.2% of the general adult population in the USA and the UK. However, only 12.3% of individuals with NCGS followed a gluten‐free diet and restriction of multiple other food products was common. NCGS was rarely an isolated phenomenon, as it frequently overlapped with DGBI and disordered eating. The presence of these comorbidities was associated with a significant increase in psychological distress, somatisation, healthcare utilisation, and reduced physical and mental quality of life.

Our estimated prevalence of self‐reported NCGS in the general population aligns with the findings of our recent global systematic review and meta‐analysis, which reported a pooled prevalence of self‐reported NCGS of 10.3% (95% CI, 7.0–14.0)^2^. Our findings are comparable to the 13% prevalence reported in a 2012 population‐based survey in the UK, but significantly lower than the 32.8% prevalence observed in a subsequent survey in 2015 [15, 16]. Conversely, the prevalence rate in our study is more than double the 5.1% prevalence previously reported in the USA [17]. Interpreting these historical discrepancies is difficult, given the substantial methodological heterogeneity noted in previous meta‐analysis [2]. Nonetheless, we mitigated these limitations by using a standardised and homogeneous methodology across both nations to provide a more reliable benchmark for current prevalence estimates.

The self‐reported NCGS cohort in our study was predominantly female, with a median age of 39 years, and reported a substantial burden of both gastrointestinal and extra‐intestinal symptoms. Bloating, abdominal pain, and fatigue were the hallmark complaints. This clinical profile is consistent with the characteristic phenotypes of self‐reported NCGS in the published literature [1, 2, 18]. Furthermore, this physical burden was compounded by significant psychological comorbidities. Similar to previous reports, individuals with self‐reported NCGS were significantly more likely to report clinically significant anxiety and depression than those without gluten sensitivity [2].

The association and symptom overlap between self‐reported NCGS and IBS is well‐documented [2, 18, 19]. Our findings confirm this strong association but demonstrate that it is not unique to IBS. We found that individuals with self‐reported NCGS had a significantly higher prevalence of several DGBIs across all assessed anatomical regions, compared with those without self‐reported NCGS. Importantly, this widespread association remained significant even after adjusting for mood disorders. This suggests that the extensive overlap between NCGS and DGBI is not a byproduct of shared psychosomatic distress but is a reflection of a generalised dysregulation of the gut‐brain axis involving both peripheral and central mechanisms.

Our results confirm that self‐reported NCGS rarely occurs in isolation and is more commonly part of a broader phenotype characterised by sensitivity to multiple foods [19, 20]. Individuals with self‐reported NCGS also reported high frequencies of sensitivity to dairy, onions, and sugar, suggesting that symptoms in this cohort may be driven by fermentable carbohydrates (FODMAPs), such as lactose, fructans and fructose, rather than gluten specifically. Previous trials suggest that symptoms in this cohort are often driven by FODMAPs like fructans, rather than gluten specifically [21, 22]. However, the substantial reporting of sensitivities to non‐fermentable proteins (e.g., eggs, fish) in our cohort indicates that FODMAPs alone cannot fully explain the symptom burden. The wide‐ranging pattern of self‐reported intolerances is likely due to heightened visceral hypersensitivity and dietary hypervigilance, whereby the anticipation of symptoms leads to the exclusion of multiple staple foods and increases the risk of nutritional deficiencies.

A concerning finding in our study was the substantial burden of disordered eating symptoms among individuals with self‐reported NCGS. We found that 31.5% of individuals with self‐reported NCGS screened positive for a probable eating disorder (SCOFF ≥ 2), and over one‐third screened positive for ARFID symptoms. These estimates are consistent with the 34.6% prevalence of ARFID symptoms we observed in the overall DGBI population within this same dataset [23]. These findings support the notion that self‐reported NCGS is not a distinct pathological entity, but a DGBI phenotype where the “fear of aversive consequences”, such as pain or bloating, is perceived to be related to gluten‐based products [7]. The discordance between perceived gluten sensitivity and actual dietary behaviour suggests that, for most individuals with self‐reported NCGS, a gluten‐free diet rarely results in sustained symptomatic improvement sufficient to justify the high financial cost and social burden of strict adherence [24]. Notably, even among those who report long‐term adherence to a gluten‐free diet, the majority continue to experience persistent gastrointestinal and extra‐intestinal symptoms [25].

Another novel finding in our study is the identification of four distinct clinical phenotypes, defined by the intersection between self‐reported NCGS, DGBI, and ARFID symptoms. Individuals with self‐reported NCGS who met the Rome IV criteria for a DGBI and were screened positive for ARFID symptoms had the highest frequencies of elevated psychological distress, somatisation, healthcare utilisation, and lower quality of life compared with the other clinical phenotypes. This aligns with large‐scale epidemiological data demonstrating that the overlap of multiple DGBI and the involvement of multiple anatomical regions is associated with increased psychological comorbidity, overall disease severity and poorer quality of life [26, 27]. Conversely, those who reported gluten sensitivity without overlapping DGBI or ARFID symptoms had a mild clinical phenotype with lower levels of psychological distress than the general population and average healthcare utilisation.

Our findings support a fundamental shift in the diagnosis and management of self‐reported NCGS, moving away from a sole focus on gluten avoidance towards a broader biopsychosocial model. Gastroenterologists must be vigilant of the fact that the profound symptom burden in this population is often driven by the cumulative effect of overlapping DGBI and disordered eating. While excluding coeliac disease and other organic conditions remains the prerequisite first step in diagnosis, routine screening for DGBI and disordered eating symptoms is necessary to identify high‐risk phenotypes. Management should include a multidisciplinary approach to reintroduce foods and challenge maladaptive avoidance behaviours, and gut‐brain directed therapies such as neuromodulators or cognitive behavioural therapy to treat the underlying central sensitisation and visceral hypersensitivity [28, 29].

The heterogeneity we have described has implications for future research, particularly gluten‐challenge trials and mechanistic studies. Randomised placebo‐controlled trials have repeatedly shown high nocebo response rates, a phenomenon largely caused by negative expectancy and hypervigilance [4, 5]. Similarly, mechanistic studies have consistently failed to identify a reproducible biomarker of immune activation or barrier dysfunction to distinguish self‐reported NCGS from other functional disorders [30]. However, these studies have treated NCGS as a monolithic clinical entity and did not account for the profound phenotypic variation we have identified. Any genuine biological signal of a specific gluten reaction may have been obscured by the “noise” of visceral hypersensitivity and psychosomatic distress inherent to the overlapping DGBI and ARFID symptoms phenotypes. This highlights the need to develop and validate diagnostic criteria for NCGS beyond the binary classification of self‐reported sensitivity. Such criteria could follow the Rome framework to establish a positive diagnosis based on specific symptom clusters rather than exclusion alone. The standardisation of diagnosis would be instrumental in guiding recruitment in future clinical trials.

To our knowledge, this is the first study to systematically explore the association between self‐reported NCGS, DGBI and disordered eating using validated diagnostic instruments, including the full Rome IV diagnostic questionnaire, NIAS and SCOFF. Another key strength was the large population sample from two countries (the USA and UK), based on nationally representative demographics. Furthermore, we performed multivariable analyses to adjust for psychological confounders to demonstrate that the association between self‐reported NCGS and DGBI persists independent of psychological distress, challenging the perception that these symptoms are purely psychosomatic. We have also provided novel data on the impact of these comorbidities on healthcare utilisation and quality of life.

Our study also had several limitations. First, the cross‐sectional design limits causal and mechanistic interpretation; we cannot ascertain whether gluten sensitivity precipitates the development of DGBI and ARFID symptoms or whether pre‐existing DGBI or disordered eating leads individuals to misattribute their symptoms to gluten. Second, the reliance on self‐reported data carries inherent risks of misclassification bias. However, the prevalence of self‐reported coeliac disease in our cohort aligns with the established global prevalence, supporting the validity of the self‐reported data [31]. Third, the study was conducted in two high‐income Western countries, which may limit the generalisability of our findings to other countries where the cultural context of gluten and the availability of gluten‐free products differ significantly. Fourth, selection bias cannot be excluded, as the internet survey followed the same methodology as the Rome Foundation Global Epidemiological Survey, and data on invited participants who did not complete the survey were not available. Finally, caution should be exercised when extrapolating these population‐based data to individuals referred to specialist gastroenterology services.

In conclusion, we found that self‐reported NCGS affects approximately one in seven adults in the USA and the UK. It is strongly associated with female sex, multiple DGBI, psychological distress, somatic symptom reporting, and disordered eating behaviours. Individuals with overlapping DGBI and ARFID symptoms represented the most severe clinical phenotype with significantly higher healthcare utilisation and poorer quality of life compared with those without these comorbidities. These findings suggest that self‐reported NCGS is a food‐related phenotype of DGBI driven by cumulative physiological and psychological burden, rather than a discrete biological hypersensitivity to gluten. Management should be multidisciplinary, focussing on dietary expansion and gut‐brain directed therapies to address the gastrointestinal, psychological and eating disorder‐related symptoms.

## Author Contributions

Conception: M.G.S., O.P., I.A. Statistical analysis and data visualisation: M.G.S. Initial drafting of the manuscript: M.G.S and I.A. Data interpretation, critical revision of the manuscript and final approval of the submitted version: M.G.S., D.S.S., H.B.‐M., M.S., O.P., I.A.

## Funding

The survey was funded by Tillotts Pharma and Novonesis. The funders played no role in study design, data collection, data analysis, data interpretation or writing of the report.

## Conflicts of Interest

M.G.S. is a Trainee Editor at UEG Journal and received speaker fees from Thermo Fisher. D.S.S. receives an educational charitable grant from Dr Schaer. H.B.‐M. receives royalties from Oxford University Press and Cambridge University Press for the sale of her books. M.S. is a Consultant for Danone Nutricia Research, Biocodex, Tillotts, Takeda, Kyowa Kirin, Abbvie, BioGaia, Renapharma, AlfaSigma, and Cinclus Pharma; received speaker fees from Tillotts, Kyowa Kirin, Takeda, Biocodex, Sanofi, Abbvie, Janssen Immunology, Pfizer, BioGaia, Renapharma, Mayoly and Bromatech; received unrestricted research grants from Genetic Analysis AS, BioGaia. OSP has no conflicts of interest to declare. I.A. received speaker fees from PrecisionBiotics and the Rome Foundation. None of these organisations was involved in the data analysis or write‐up of this study.

## Supporting information


Supporting Information S1

**Table 1:** Baseline characteristics of study participants by country.

## Data Availability

The data that support the findings of this study are available from the corresponding author upon reasonable request.
