# Supplementary material for: Prevalence of Self‐Reported Non‐Coeliac Gluten Sensitivity and Its Association With Disorders of Gut‐Brain Interaction and Disordered Eating
Source: United European Gastroenterol J. 2026 Jul 3;14(6):e70256. doi: 10.1002/ueg2.70256 (PMC13329839; doi:10.1002/ueg2.70256)
Supplement: Supplementary file 1 — Supporting Information S1 [file UEG2-14-e70256-s002.docx]

# The following validated instruments were used to assess clinical variables:

## Rome IV diagnostic questionnaire

We used the complete Rome IV diagnostic questionnaire for adults to identify DGBI. Diagnoses were categorised according to the six anatomical regions defined by the Rome diagnostic system: oesophageal, gastroduodenal, bowel, anorectal, biliary and centrally mediated disorders of gastrointestinal pain. Symptom frequencies derived from the Rome IV questionnaire were dichotomised based on established clinical thresholds. Symptoms were coded as “present” if they met the minimum frequency criteria required for a Rome IV diagnosis (e.g., abdominal pain occurring ≥1 day/week; diarrhoea occurring in >20% of bowel movements).

## Nine-Item ARFID screen (NIAS)

We used this validated questionnaire to assess restrictive eating habits. It comprises nine statements rated on a scale from 0 (“strongly disagree”) to 5 (“strongly agree”), yielding a total score out of 45. The NIAS includes three sub-scales mapping to specific ARFID phenotypes: NIAS-Picky (sensory-based avoidance, cut-off ≥10), NIAS-Interest (lack of interest in eating/appetite, cut-off ≥9), and NIAS-Fear (fear of aversive consequences, cut-off ≥10). Internal consistency in our sample was high for all subscales as indicated by Cronbach’s alpha: 0.825 for NIAS-Picky, 0.844 for NIAS-Appetite, and 0.899 for NIAS-Fear.

## SCOFF eating disorder screen

This validated 5-item questionnaire was used to screen for core features of anorexia nervosa and bulimia nervosa. Respondents answered “yes” or “no” to five questions addressing self-induced vomiting, loss of control over eating, significant recent weight loss, body image distortion, and the extent to which food dominates their lives. Each affirmative response scores 1 point (total range 0-5), with a total score of ≥2 indicating a positive screen for a potential eating disorder.

## Patient Health Questionnaire-4 (PHQ-4)

We evaluated psychological distress using the PHQ-4. This validated 4-item instrument assesses the frequency with which participants have been bothered by anxiety and depression symptoms over the preceding two weeks, from 0 (“not at all”) to 3 (“nearly every day”). Based on two questions for anxiety and two questions for depression, a total score of ≥3 on the anxiety or depression subscales indicates clinically significant symptoms.

## Patient Health Questionnaire-12 (PHQ-12)

This is a modified version of the PHQ-15 that excludes gastrointestinal symptoms (nausea, abdominal pain, altered bowel habits) to provide more accurate somatisation scores for individuals with DGBI. Participants rated how much they were bothered by 12 somatic symptoms (back pain, pain in their arms, legs or joints, headaches, chest pain, dizziness, fainting spells, heart pounding or racing, shortness of breath, pain or problems during sexual intercourse, trouble sleeping, and menstrual cramps or problems with periods) over the preceding 4 weeks on a scale of 0 (“not bothered at all”) to 2 (“bothered a lot”). Higher scores indicate greater distress around somatic symptoms, with 4 severity levels: minimal (0-3), low (4-7), medium (8-12), and high (≥13).

## PROMIS Global-10 quality of life (QOL)

We evaluated health-related QOL using this validated 10-item instrument, which assesses the following domains: general health, quality of life, mental and physical health, social activities, relationships and roles, ability to carry out physical activities, emotional distress, fatigue and pain. Raw scores were converted into T-scores with standard errors, where a score of 50 represents the population’s mean. Scores were dichotomised as <50 (below average) and ≥50 (above average).
