# Supplementary material for: Prevalence of Self‐Reported Non‐Coeliac Gluten Sensitivity and Its Association With Disorders of Gut‐Brain Interaction and Disordered Eating
Source: United European Gastroenterol J. 2026 Jul 3;14(6):e70256. doi: 10.1002/ueg2.70256 (PMC13329839; doi:10.1002/ueg2.70256)
Supplement: Supplementary file 2 — Table 1: Baseline characteristics of study participants by country. [file UEG2-14-e70256-s001.docx]

**Supplementary Table 1 – Baseline characteristics of study participants by country**

|  | USA (n=2000) | UK (n=2002) |
| --- | --- | --- |
| Female sex, n (%) | 1000 (50.0) | 1000 (50.0) |
| White ethnicity, n (%) | 1518 (75.9) | 1728 (86.3) |
| Age categories, n (%) |  |  |
| 18 – 39 years | 798 (39.9) | 798 (39.9) |
| 40 – 64 years | 801 (40.1) | 805 (40.2) |
| ≥65 years | 401 (20.1) | 399 (19.9) |
